# Symptom Recovery in Children Aged 5 to 12 Years With Sport-Related and Non–Sport-Related Concussion

**DOI:** 10.1001/jamanetworkopen.2024.48797

**Published:** 2024-12-04

**Authors:** Andrée-Anne Ledoux, Veronik Sicard, Vid Bijelic, Nick Barrowman, Jacqueline van Ierssel, Darcy Beer, Kathy Boutis, Emma Burns, William Craig, Stephen B. Freedman, Isabelle Gagnon, Jocelyn Gravel, Gurinder Sangha, Keith Owen Yeates, Martin Osmond, Roger Zemek

**Affiliations:** 1 Children’s Hospital of Eastern Ontario Research Institute, Ottawa, Ontario, Canada; 2 Department of Cellular Molecular Medicine, University of Ottawa, Ottawa, Ontario, Canada; 3 The Children’s Hospital Research Institute of Manitoba, Pediatric Emergency Department, Winnipeg, Manitoba, Canada; 4 Division of Paediatric Emergency Medicine, Hospital for Sick Children, Toronto, Ontario, Canada; 5 Division of Pediatric Emergency Medicine, IWK Health Centre, Halifax, Nova Scotia, Canada; 6 Stollery Children’s Hospital, Pediatric Emergency, Edmonton, Alberta, Canada; 7 Department of Pediatrics, Alberta Children’s Hospital, Pediatric Emergency, Calgary, Alberta, Canada; 8 Department of Pediatrics, Montreal Children’s Hospital, Montreal, Quebec, Canada; 9 Hospital Sainte-Justine, Pediatric Emergency Medicine Department, Montreal, Quebec, Canada; 10 Children’s Hospital London Health Sciences Centre, Western University, London, Ontario, Canada; 11 Department of Psychology, Alberta Children’s Hospital Research Institute, and Hotchkiss Brain Institute, University of Calgary, Calgary, Alberta; 12 Department of Pediatrics, University of Ottawa, Ottawa, Ontario, Canada; 13 Department of Emergency Medicine, University of Ottawa, Ottawa, Ontario, Canada

## Abstract

**Importance:**

There is limited research on clinical features and symptom recovery from sport-related concussion (SRC) and non-SRC in younger children.

**Objective:**

To investigate the trajectory of symptom recovery in children aged 5 to 7 years and 8 to 12 years with SRC and non-SRC at 1, 2, 4, 8, and 12 weeks postinjury.

**Design, Setting, and Participants:**

This secondary analysis of a prospective multicenter cohort study (Predicting and Preventing Postconcussive Problems in Pediatrics) included participants aged 5 to 12 years with acute concussion who presented to 9 pediatric emergency departments within the Pediatric Emergency Research Canada network between August 2013 and June 2015. Analyses were conducted from September 2023 to May 2024.

**Exposure:**

Participants had a concussion consistent with the Zurich consensus diagnostic criteria and 85% completeness of the Post-Concussion Symptom Inventory (PCSI) at each time point.

**Main Outcomes and Measures:**

The primary outcome was symptom change, defined as current minus preinjury ratings 1, 2, 4, 8, and 12 weeks postinjury, measured using the PCSI. Symptoms were self-rated for children aged 8 to 12 years and child- and parent-rated for children aged 5 to 7 years. Mixed-effect models were conducted using total PCSI score adjusting for random effects; fixed-effect indicators included injury setting (SRC and non-SRC), time, injury setting × time, and other variables associated with recovery.

**Results:**

A total of 1747 children, including 513 aged 5 to 7 years (mean [SD] age, 6.57 [0.85] years; 320 male [62.4%]) and 1234 aged 8 to 12 years (mean [SD] age, 10.68 [1.40] years; 806 male [65.3%]) were recruited, of whom 477 aged 5 to 7 years and 1157 aged 8 to 12 years were included in the analysis. Of those included in the analysis, 207 aged 5 to 7 years (43.4%; mean [SD] age, 6.68 [0.84] years; 142 male [68.6%]) and 790 aged 8 to 12 years (67.2%; mean [SD] age, 10.77 [1.40] years; 547 male [69.2%]) sustained an SRC. No significant differences in recovery curves across time postinjury were found between those with SRC and non-SRC (5-7 years: β = −0.09; 95% CI, −1.10 to 0.92; 8-12 years: β = 0.11; 95%CI, −1.50 to 1.70).

**Conclusions and Relevance:**

In this cohort study of children aged 5 to 12 years with an acute SRC or non-SRC, symptom recovery trajectories over time were similar in both groups. This finding suggests similar management protocols can be used for sport and nonsport mechanisms of injury (excluding assault and motor vehicle crash) in the younger population.

## Key Points

**Question:**

Are symptom recovery trajectories similar between children who have sustained a sport-related concussion vs a non–sport-related concussion?

**Findings:**

In this cohort study of 1747 children aged 5 to 12 years with an acute concussion, there were no differences in symptom recovery between sport-related concussions and non–sport-related concussions and between contact and collision, limited-contact or limited-impact, and noncontact sports.

**Meaning:**

These findings suggest that children aged 5 to 7 and 8 to 12 years have similar symptom recovery trajectories across time regardless of the injury mechanism and that sport and nonsport injuries should be managed similarly.

## Teaser

This cohort study assesses symptom recovery over time among children in Canada with sport-related and non–sport-related concussion.

## Introduction

Each year, an estimated 50 to 60 million people worldwide experience traumatic brain injuries (TBIs), with TBIs or concussions accounting for 75% to 90% of hospital cases.^1,2^ Pediatric concussion represents an important health concern worldwide; both the Berlin and Amsterdam Consensus Statements on Concussion in Sport highlighted the critical need for more research specifically focused on the 5- to 12-year age group,^3,4,5^ emphasizing the necessity for high-quality, age-specific studies with minimal bias to better understand prognosis in this demographic.^6,7,8^ Despite the plethora of pediatric concussion studies, most have focused on adolescents and young adults^9,10,11^ with sport-related concussion (SRC).^10,12,13^ An improved understanding of the characteristics of concussion sustained in in children aged 5 to 12 years, both in sport and nonsport contexts, could influence clinical management of concussions.

The developing brain may be more vulnerable to injury.^14,15^ Different neurodevelopmental stages likely play a pivotal role in concussive injury and recovery mechanisms because the maturity of neural tissue and the structure of the brain in relation to the skull and its musculature evolve throughout development.^15,16^As such, different mechanisms of injury may impact younger children differently than older children and influence clinical and neurophysiological recovery trajectories.

By 6 years of age, approximately one-half of concussions are attributed to sports and recreational activities, and the proportion steadily rises to approximately 80% by 10 years of age and remains consistent until 16 years of age.^6,7,8^ While injury mechanisms are well-defined for the overall pediatric population,^6^ they have not been adequately stratified by age group. Furthermore, limited research has focused on recovery in children aged 5 to 12 years. Although a large cohort study^17^ observed that younger children (aged 5-7 years and 8-12 years) tend to recover more rapidly compared with older age groups, uncertainties persist regarding whether the recovery trajectories of those with SRC are similar to those experiencing non-SRC. Previous research on concussion injury and recovery in children is based on combined samples of children and adolescents,^18^ which may confound age-specific trends. Furthermore, studies have often merged mechanisms of injury^13,17^ or exclusively focused on SRC,^10,12^ utilized retrospective designs,^12,13^ and have had limited sample sizes.^9,19,20^

Given the diverse settings in which physical and sports activities occur in younger children, ranging from organized sports to informal play during recess or gym classes, it is important to study pediatric concussion across various settings to comprehensively characterize recovery in these age groups. For the purposes of this study, we defined sports broadly to encompass any form of organized or casual physical activity. The objectives of this study were to (1) describe a prospective cohort of children aged 5 to 12 years that sustained an SRC or non-SRC, outlining patient demographics, premorbid conditions, concussion signs and symptoms, and mechanisms of sports injury for those with an SRC; (2) investigate symptom recovery trajectories in children with SRC and non-SRC over time; (3) examine the association of sports classification (contact or collision, limited contact or impact, and noncontact) with symptom recovery trajectories; and (4) determine the proportion of children with persisting symptoms after concussion (PSAC) in those with SRC.

## Methods

This study was a planned secondary analysis of a prospective multicenter cohort study (Predicting Persistent Postconcussive Problems in Pediatrics [5P]),^18,21^ conducted from August 2013 to May 2015 at 9 pediatric emergency departments (EDs) in the Pediatric Emergency Research Canada (PERC) network.^22^ Patients and the public contributed to the study design, conduct, and how to disseminate results.^21^ The study protocol was previously published.^21^ This study was conducted and reported under the Strengthening the Reporting of Observational Studies in Epidemiology (STROBE) reporting guideline for cohort studies. The ethics committees of the participating 9 institutions approved the study, and this secondary analysis was approved by the Children’s Hospital of Eastern Ontario Research Ethics Board. All participants provided written informed consent or assent.

### Equity, Diversity, and Inclusion

The author team was gender balanced and included junior, mid-career, and senior researchers from multiple disciplines. The overall 5P study population included a spectrum of individuals of different ages (5-18 years), genders, demographics, and comorbidities from across Canada.

### Participants

To be included in this substudy, participants were required to be aged 5 to 12 years and present within 48 hours of injury to a PERC study ED with acute SRC or non-SRC consistent with the diagnostic criteria of the Zurich Consensus Statement on Concussion in Sport.^23^ Exclusion criteria included Glasgow Coma Scale score of 13 or less; any trauma-related abnormality on neuroimaging; neurosurgical intervention, intubation, or intensive care unit admission; multisystem injury requiring hospitalization; severe preexisting neurological developmental delay resulting in communication difficulties; intoxication; absence of trauma as a primary event; previously enrolled; language barrier; or inability to complete follow-up. Patients with structural neuroimaging abnormalities (eg, small subdural hemorrhages, subarachnoid hemorrhages, or small intraparenchymal hemorrhages) were excluded, even if the Glasgow Coma Scale score was normal. The absence of lesions on standard structural neuroimaging is required to meet the definition of concussion,^5^ and the presence of a structural injury may signify a more serious TBI.^24^ Patients who sustained a second concussion after enrollment during the study period were excluded from the analyses.

Children were classified as having an SRC if their concussion occurred while playing a sport of any kind, including organized team sports or recreational sports and recess sports. See eTable 1 in Supplement 1 for examples. Following the American Academy of Pediatrics classification,^25^ sports were classified as contact or collision, limited contact or impact, and noncontact. See eTable 2 in Supplement 1 for classification.

Children were classified as having a non-SRC if their concussion occurred due to another mechanism of injury than sport, such as falls outside sport or recreation settings. See eTable 1 in Supplement 1 for examples. Those injured in motor vehicle crashes or assaults were not included in our analysis.

### ED Visit

Once enrolled, demographic and medical history information was collected via a Research Electronic Data Capture (REDCap)^26^ survey. The number of school days missed (0, 1-2, 3-6, and ≥7 days) for any reason over the last 6 months was used as an indicator of baseline functional status.

Participants underwent comprehensive evaluations utilizing 3 distinct tools. The first tool was the Acute Concussion Evaluation (ACE),^27^ a standardized tool encompassing (1) injury characteristics, such as mechanism of injury, loss of consciousness, amnesia, early signs of concussion (eg, dazed or confused, answers questions slowly, repeats questions, or forgetful), and seizures; (2) a 22-item dichotomous symptom inventory designed to assess injury characteristics, symptoms; and (3) assessment of risk factors associated with prolonged recovery. The second tool was the Child-Sport Concussion Assessment Tool 3 (Child-SCAT3),^28^ a standardized tool focusing on cognitive assessment and balance. The third tool was the Post-Concussion Symptom Inventory (PCSI),^29^ a validated and reliable tool encompassing (1) a 20-item, 7-point scale for parent-reported symptoms, with a score range of 0 to 120, and (2) developmentally specific self-report forms for children aged 5 to 7 years with a 13-item, 3-point scale (0-2), with a score range of 0 to 26, and children aged 8 to 12 years with a 17-item, 3-point scale (0-2), with a score range of 0 to 34. Higher scores on the PCSI indicate an increased symptom severity. Additionally, participants or their parents provided details on the mechanism of the traumatic event (eg, type of sport or whether a helmet or mouthguard was worn) and shared information about personal and mental health history.

### Follow-Up Questionnaires

Using the PCSI, symptoms were self-rated for children aged 8 to 12 years and child- and parent-rated for children aged 5 to 7 years at presentation and 1, 2, 4, 8, and 12 weeks postinjury. Data were entered into REDCap^26^ database.

### Outcomes

The primary outcome was symptom change, defined as current ratings minus preinjury ratings (Δ score), across time (1, 2, 4, 8, and 12 weeks), measured using the PCSI. An 85% completeness of the PCSI items at each time point was required. The secondary outcome was PSAC, defined as 3 or more new or worse symptoms (ie, 3 PCSI items with a positive Δ score).

### Statistical Analysis

Statistical analyses were conducted from September 2023 to May 2024 using R Statistical Software version 4.3.1 (R Project for Statistical Computing),^30^ with key packages including lme4 versions 1.1 to 35.1,^31^ rms version 6.7-1,^32,33^ and emmeans version 1.10.0.^34^ Baseline characteristics were compared descriptively between the 2 age groups (children aged 5-7 years and 8-12 years) by injury setting (SRC and non-SRC) using 95% CIs.

The primary analysis evaluated symptom recovery over 12 weeks, stratified by injury setting (SRC or non-SRC). Other factors were selected based on previous 5P work,^17,18^ other research, and perceived clinical usefulness. They included sex, age group, time, injury setting, injury setting × time, previous concussion with symptoms lasting longer than 1 week, migraine history, history of mental health problems (learning disability, attention deficit disorder, anxiety, and depression), school days missed over the past 6 months for any reason, injury characteristics (loss of consciousness and duration, seizure following injury, dazed or confused, answers questions slowly, repeats questions, forgetful, total cognitive scores from the Child-SCAT3, and tandem stance on the Balance Error Scoring System [BESS]), sports classification (ie, contact or collision, limited contact or impact, and noncontact), and sport characteristics (wore a helmet or wore a mouthguard). Two linear mixed-effects models (one for each age group [5-7 years and 8-12 years]) were fitted with the total PCSI Δ score as the dependent variable, measured at baseline and at 1, 2, 4, 8, and 12 weeks. To allow for nonlinearity, we used restricted cubic splines with 4 knots for age, total cognitive score, and tandem stance variables.

The secondary analysis focused on symptom recovery over 12 weeks in children with SRC, stratified by sports classification. As with the primary analysis, 2 linear mixed-effects models were fitted with the total PCSI Δ score as the dependent variable. The other factors were the same as those used in the primary analysis.

For all analyses, children were removed from the analyses if they had any missing data for covariates or missing more than 15% of PCSI data at all follow-up time points. eTable 3 in Supplement 1 presents the percentage of missing PCSI outcomes at each time point.

The proportion of those with PSAC was described at each study follow-up time points (1, 2, 4, 8, and 12 weeks) for both SRC and non-SRC, as well as by the sport classification. Random-effects logistic models were used to examine the association of sport classification with PSAC over time. The threshold for statistical significance was a 2-sided *P* < .05.

## Results

A total 1747 children, including 513 aged 5 to 7 years (mean [SD] age, 6.57 [0.85] years; 320 male [62.4%]) and 1234 children aged 8 to 12 years (mean [SD] age, 10.68 [1.40] years; 806 male [65.3%]) were recruited, of whom 477 aged 5 to 7 years and 1157 aged 8 to 12 years were included in the analysis (eFigure 1 in Supplement 1). Of these participants, 207 aged 5 to 7 years (43.4%; mean [SD] age, 6.68 [0.84] years; 142 male [68.6%]) and 790 aged 8 to 12 years (67.2%; mean [SD] age, 10.77 [1.40] years; 547 male [69.2%]) sustained an SRC (Table).

**Table.** Participant Characteristics

Variable	Total study sample, No. (%) (N = 1749)		Analyzed sample, No. (%) (N = 1634)			
	Age 5-7 y (n = 513)	Age 8-12 y (n = 1234)	Age 5-7 y (n = 477)		Age 8-12 y (n = 1157)	
			Nonsport or fall (n = 270)	Sports and recreation (n = 207)	Nonsport or fall (n = 367)	Sports and recreation (n = 790)
Age, mean (SD), y	6.57 (0.85)	10.68 (1.40)	6.54 (0.83)	6.68 (0.84)	10.54 (1.35)	10.77 (1.40)
Sex						
Male	320 (62.4)	806 (65.3)	157 (58.1)	142 (68.6)	214 (58.3)	547 (69.2)
Female	193 (37.6)	428 (34.7)	113 (41.9)	65 (31.4)	153 (41.7)	243 (30.8)
Time from concussion to triage, mean (SD), h	6 (9)	8 (12)	7 (9)	6 (9)	8 (13)	8 (11)
Sport classification^a^						
Noncontact	411 (80.1)	583 (47.2)	270 (100)	111 (53.6)	367 (100)	176 (22.3)
Limited contact or impact	46 (9.0)	246 (19.9)	0	44 (21.3)	0	229 (29.0)
Contact or collision	54 (10.5)	399 (32.3)	0	52 (25.1)	0	385 (48.7)
Prior concussion and symptom duration						
No prior concussion (symptom duration <1 wk)	496 (96.7)	1141 (92.5)	262 (97.0)	201 (97.1)	339 (92.4)	736 (93.2)
Prior concussion (symptom duration ≥1 wk)	14 (2.7)	89 (7.2)	8 (3.0)	6 (2.9)	28 (7.6)	54 (6.8)
Personal history of migraine	24 (4.7)	125 (10.1)	13 (4.8)	10 (4.8)	42 (11.4)	76 (9.6)
History of learning disability	21 (4.1)	96 (7.8)	9 (3.3)	9 (4.3)	31 (8.4)	57 (7.2)
History of attention-deficit/hyperactivity disorder	26 (5.1)	111 (9.1)	8 (3.0)	14 (6.8)	32 (8.7)	71 (9.0)
History of anxiety	12 (2.3)	80 (6.5)	4 (1.5)	7 (3.4)	27 (7.4)	49 (6.2)
History of depression	0	1 (0.1)	0	0	4 (1.1)	9 (1.1)
Loss of consciousness duration, mean (SD), min	0.08 (0.51)	0.11 (0.62)	0.03 (0.17)	0.16 (0.76)	0.18 (0.84)	0.08 (0.50)
Seizure following injury	10 (2.0)	23 (1.9)	4 (1.5)	5 (2.4)	6 (1.6)	14 (1.8)
Acute Concussion Evaluation						
Appears dazed and confused	193 (37.6)	23 (1.9)	102 (37.8)	81 (39.1)	162 (44.1)	390 (49.4)
Answers questions slowly	179 (34.9)	584 (47.3)	94 (34.8)	71 (34.3)	138 (37.6)	328 (41.5)
Repeats questions	57 (11.1)	500 (40.5)	29 (10.7)	24 (11.5)	42 (11.4)	92 (11.6)
Forgetful of recent information	84 (16.4)	146 (11.8)	47 (17.4)	30 (14.5)	71 (19.3)	140 (17.7)
Wore helmet	26 (5.1)	225 (18.2)	0	26 (12.5)	0	320 (40.5)
Wore mouthguard	10 (2.0)	151 (12.2)	0	10 (4.8)	0	145 (18.4)
No. of school days missed over past 6 mo for any reason						
0	116 (22.6)	227 (18.4)	62 (22.9)	47 (22.7)	80 (21.8)	136 (17.2)
1-2	193 (37.6)	441 (35.7)	105 (38.9)	77 (37.2)	108 (29.4)	270 (34.1)
3-6	122 (23.8)	365 (29.6)	58 (21.5)	57 (27.5)	101 (27.5)	254 (32.2)
≥7	76 (14.8)	225 (18.2)	45 (16.7)	26 (12.6)	78 (21.2)	130 (16.5)
Child-SCAT3 cognitive score (total), mean (SD)	18.6 (6.0)	24.9 (3.4)	18.5 (6.1)	19.1 (5.6)	24.7 (3.6)	25.0 (3.2)
BESS tandem stance No. of errors, mean (SD)	4.8 (3.8)	3.8 (3.6)	5.0 (3.8)	4.6 (3.7)	4.0 (3.7)	3.8 (3.6)
Normal neck range, No./total No. (%)	420/442 (95.0)	1048/1078 (97.2)	228/240 (95.0)	163/173 (94.2)	316/321 (88.4)	676/696 (97.1)
Tenderness of neck, No./total No. (%)	46/440 (10.5)	211/1076 (19.6)	22/239 (9.2)	20/173 (11.6)	51/322 (15.8)	147/695 (21.2)

Abbreviations: BESS, Balance Error Scoring System; SCAT3, Sport Concussion Assessment Test-3.

^a^ Per the American Academy of Pediatrics classification.

**Figure 1.**
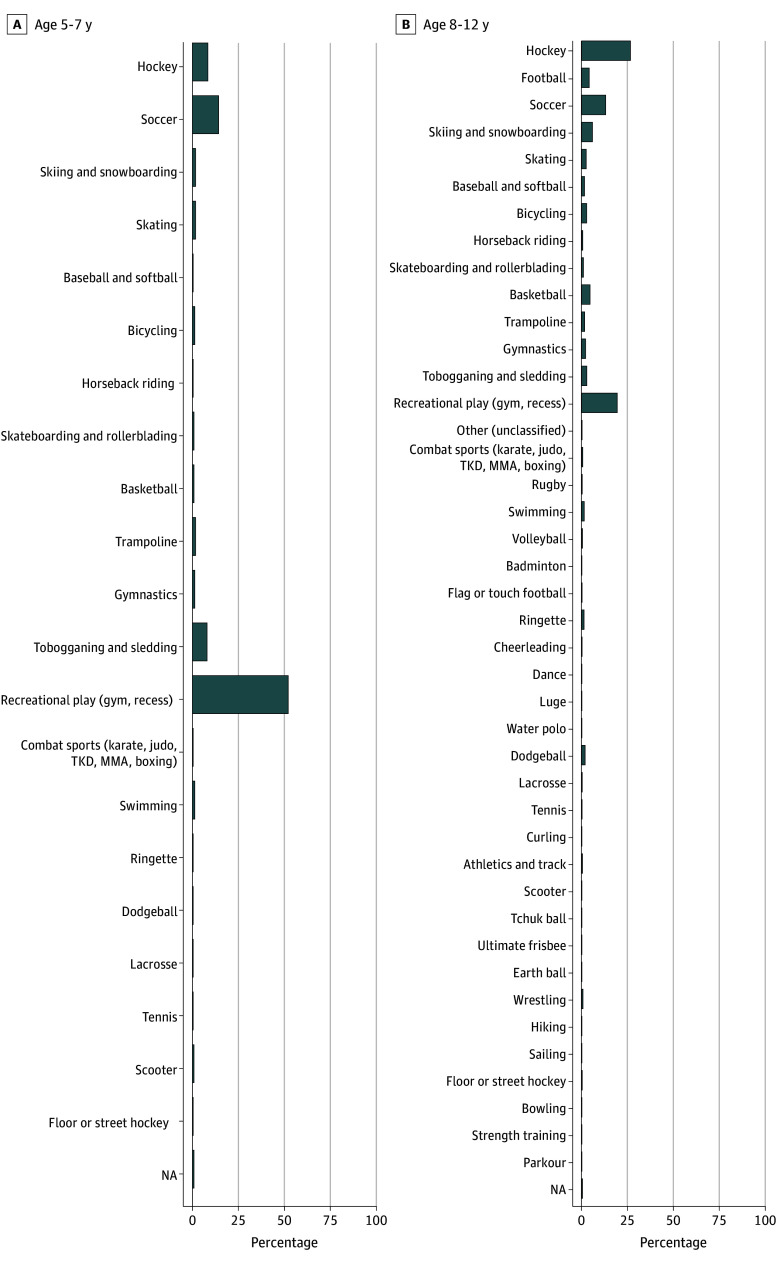
Frequency of Sport and Recreation Activities Performed at the Time of Concussion MMA indicates mixed martial arts; NA, not applicable; TKD, taekwondo.

In children aged 5 to 7 years with SRC, 111 (53.6%) sustained their SRC in noncontact sports, 44 (21.3%) in limited-contact or limited-impact sports, and 52 (25.1%) in contact or collision sports (eTable 4 in Supplement 1). The most prevalent injury settings were recreational play or recess, soccer, hockey, and tobogganing or sledding (Figure 1).

In children aged 8 to 12 years with SRC, 176 (22.3%) sustained their SRC in noncontact sports, 229 (29.0%) in limited-contact sports, and 385 (48.7%) in collision sports (eTable 4 in Supplement 1). The most prevalent injury settings were hockey, recreational play, soccer, snowboarding, basketball, and football (Figure 1).

### Association of Symptom Trajectories With Time Stratified by SRC vs Non-SRC

**Figure 2.**
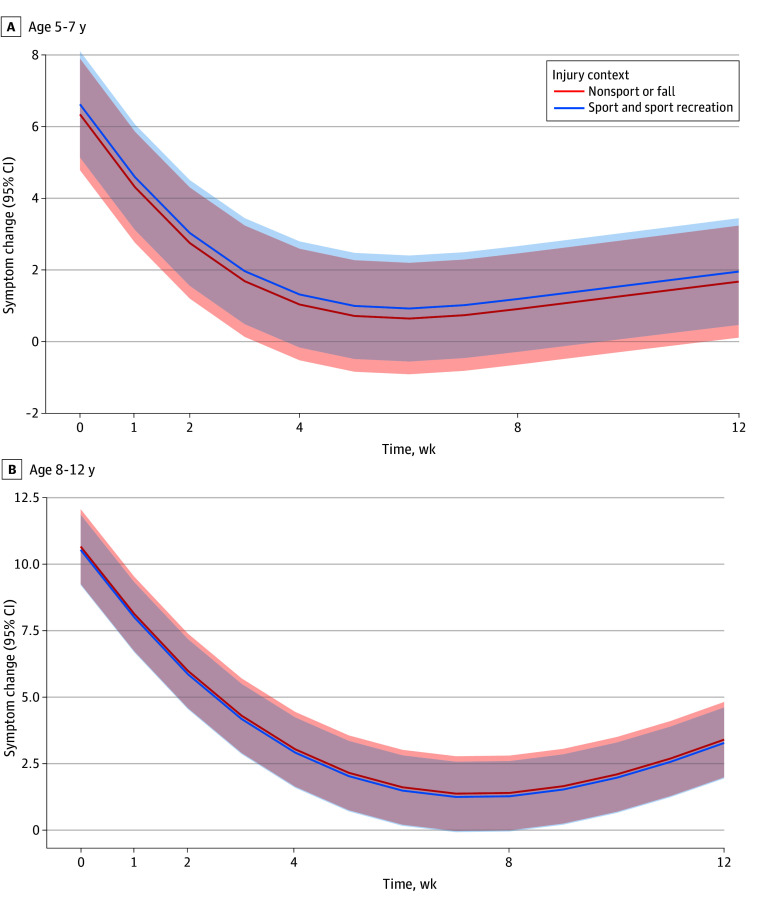
Symptom Change Across Time for Sport vs Nonsport Concussion The red line indicates nonsport concussion and the blue line indicates sport concussion. The shaded areas show the 95% CIs.

No significant differences in recovery trajectories across time postinjury were found between children with SRC and non-SRC (Figure 2). Both SRC and non-SRC showed a nonlinear association with time, with symptoms decreasing over time. For children aged 5 to 7 years, recovery was significantly associated with time (nonlinear β = 6.20; 95% CI, 5.60 to 6.90; *P* < .001). Symptom burden was positively associated with appearing dazed and confused (β = 0.68; 95% CI, 0.20 to 1.20; *P* = .01), school days missed over the past 6 months (β = 0.24; 95% CI, 0.03 to 0.45; *P* = .03), and BESS tandem stance (β = 0.06; 95% CI, 0.00 to 0.11; *P* = .04). For children aged 8 to 12 years, symptom burden was positively associated with history of anxiety (β = 1.10; 95% CI, 0.26 to 1.90; *P* = .01), loss of consciousness and duration (β = 0.41; 95% CI, 0.09 to 0.73; *P* = .01), appearing dazed and confused (β = 0.68; 95% CI, 0.24 to 1.10; *P* = .002), answering questions slowly (β = 0.78; 95% CI, 0.33 to 1.20; *P* < .001), repeating questions (β = 0.79;95% CI, 0.14 to 1.40; *P* = .02), forgetting recent information (β = 0.76; 95% CI, 0.21 to 1.30; *P* = .007), school days missed over the past 6 months (β = 0.29; 95%CI, 0.09 to 0.48; *P* = .004), and BESS tandem stance (β = 0.16; 95%CI, 0.10 to 0.21; *P* < .001). Symptom burden was negatively associated with Child-SCAT3 total cognitive score (β = −0.08; 95%CI, −0.14 to −0.02; *P* = .008). See eTable 5 and eTable 6 in Supplement 1 for full results.

### Association of Symptom Trajectories and Time Stratified by Sport Classification

**Figure 3.**
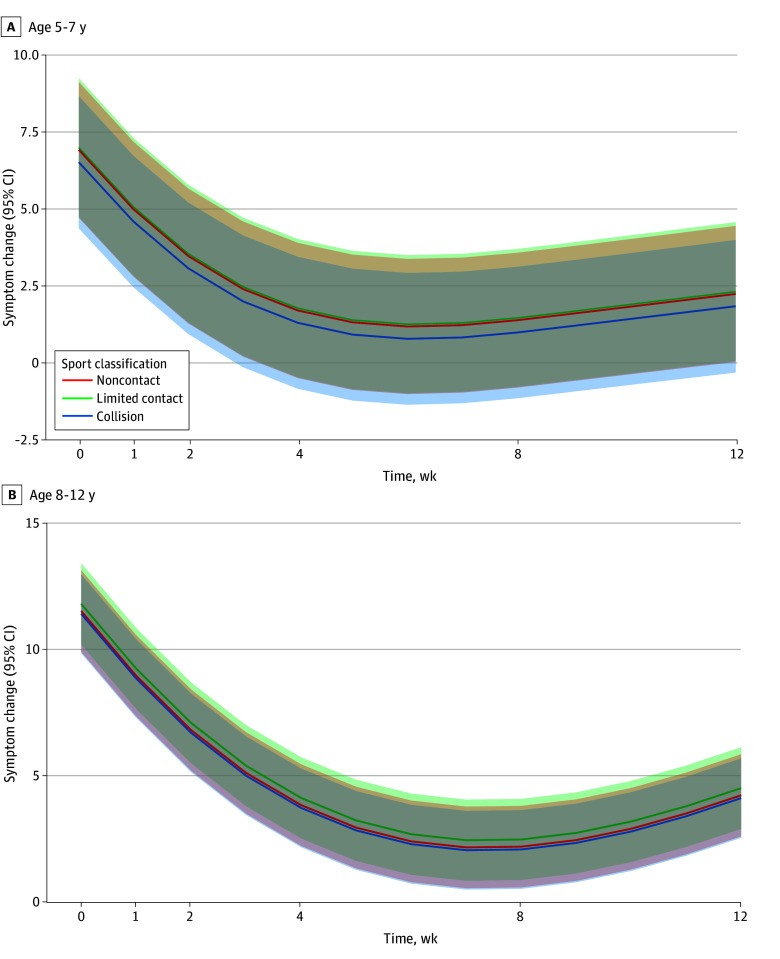
Symptom Change Across Time in Sport-Related Concussion The red line indicates noncontact sport, the green line indicates limited-contact sport, and the blue line indicates collision and contact sports. The shaded areas indicate the 95% CIs.

No significant differences in recovery curves across time postinjury were found between children with SRC and non-SRC (5-7 years:β = −0.09; 95% CI, −1.10 to 0.92; 8-12 years: β = 0.11; 95%CI, −1.50 to 1.70) (Figure 3). Symptom change across time was not associated with the sport classification (ie, contact or collision, limited contact or impact, or noncontact. See eTables 7 and eTable 8 in Supplement 1 for full results.

### Percentage of Participants With PSAC at Each Time Point by Sport Classification

**Figure 4.**
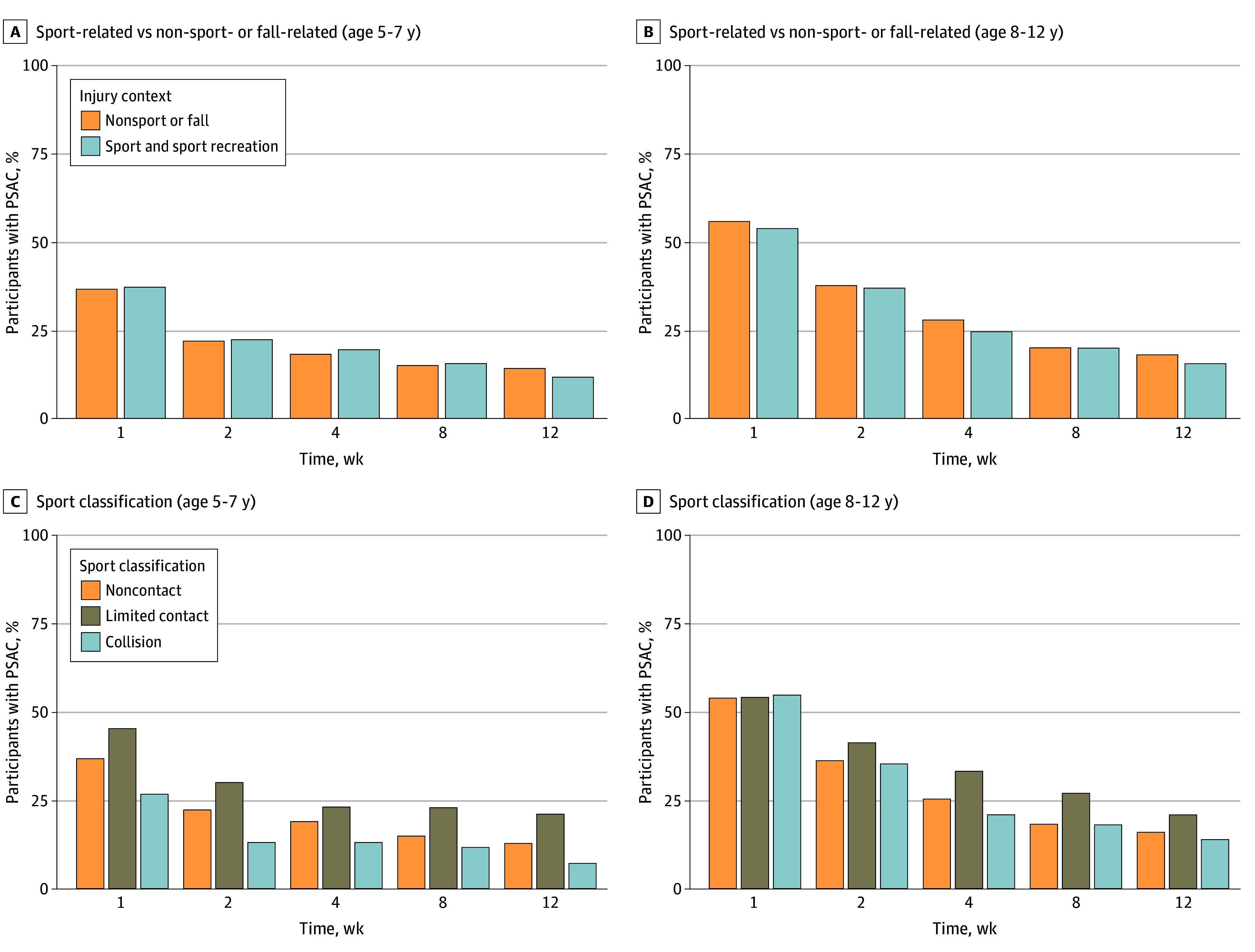
Percentage of Persisting Symptoms After Concussion (PSAC) Across Injury Contexts (Sport vs Nonsport Concussion) and Sport Classification

The percentage of participants with PSAC, stratified by SRC vs non-SRC, was similar across time for both age groups (Figure 4A and B). However, when stratified by sport classification, limited contact was associated with an increased percentage of PSAC compared with contact or collision sport (eTables 9-12 in Supplement 1). When exploring the classification of sports with more than 10 cases of PSAC by type of contact (eFigure 2 in Supplement 1), many sports with increased PSAC belonging to the limited-contact group had increased velocity or a higher possibility of falling from a greater height (ie, skiing or snowboarding, skating, bicycling, gymnastic, and tobogganing or sledding). Dodgeball had an increased percentage of PSAC across time for the 8- to 12-year-old group.

## Discussion

In this prospective cohort study involving preadolescent children, we observed no differences in symptom trajectories between children with SRC and non-SRC. Furthermore, we found no differences in symptom trajectories between different sport classifications, including noncontact, limited-contact or limited-impact, and contact or collision sports, within both age groups. Interestingly, our descriptive analysis revealed that certain sports exhibited a higher proportion of PSAC over time. Specifically, sports categorized as limited contact or impact showed an increased incidence of PSAC, but especially among those with higher velocity or a higher possibility of falling from a greater height, such as snowboarding or skiing, tobogganing or sledding, dodgeball, gymnastics and trampoline, bicycling, basketball, and baseball or softball.

Our study highlights the wide array of sports and activities in which children aged 5 to 7 years and 8 to 12 years engage. Consistent with existing research, we observed a trend where children tended to transition to more organized sports as they grew older.^6,7^ The predominant injury settings varied between the 2 age groups, with recreational play and recess activities being prominent among the younger cohort, while organized sports like hockey, soccer, and football were more prevalent among the older cohort. The sports classification revealed differences in the distribution of SRC across noncontact, limited-contact or limited-impact, and contact or collision sports in the 2 age groups. Contact or collision sports accounted for a higher percentage of SRC in the older age group compared with the younger age group. Collisions, such as those arising from bodychecking, are associated with increased risk of injury^35,36,37^ and concussion.^38,39^ Given that the brain is still developing, following the American Academy of Pediatrics and Canadian Pediatric Society guidelines, recommendations to delay bodychecking in collision sports should be respected.^40^

No significant differences in symptom trajectories were found between those with SRC and non-SRC (excluding motor vehicle crashes and assaults). Similar to previous studies, several factors were identified as significantly associated with slower recovery, including symptoms such as appearing dazed and confused, anxiety,^12,41^ loss of consciousness,^42^ and altered mental status such as answering questions slowly^18^ and forgetting recent information.^12^ Moreover, no significant differences in symptom trajectories were found between sport classifications, suggesting that symptom recovery postinjury is not influenced by the type of contact in sports-related activities for these 2 age groups. Similarly, the mechanism of injury (SRC or non-SRC) was not associated with longer recovery in a study of children presenting to a neurology clinic with the diagnosis of concussion at 73 days (on average) postinjury.^43^ However, several other studies in adolescents and adults, have found significant differences between sport and nonsport settings,^44,45,46,47^ where those with an SRC tend to recover faster than those with non-SRC. It is possible that we have not found a difference between SRC and non-SRC because we excluded motor vehicle crashes and assaults and restricted the analysis to younger children. Motor vehicle crashes are a more serious injury with a larger postinjury symptom burden than typical concussions sustained during sports.^48,49^

In the present study, the recovery trajectory in limited-contact or limited-impact sports, although not significantly different than the other trajectories, was associated with more symptoms across time. This observation seems to be due to sports with a potentially higher impact velocity or falls from greater heights, such as skiing or snowboarding, bicycling, tobogganing, gymnastics, and trampoline. A larger sample with more information on injury height and velocity would be required to confirm whether an association exists. In a large cohort of National Collegiate Athletic Association student athletes with SRC, sports classification was not associated with odds of PSAC at 4 weeks,^50^ although males in contact sports had a higher incidence of SRC compared with females, whereas this pattern was reversed for limited and noncontact sports.^51^ Our findings suggest that American Academy of Pediatrics sport classifications may not be significant clinical recovery prognosticators of concussions.

Finally, our analysis of the proportion of participants with PSAC at each time point by sports classification revealed interesting trends. While the percentage of participants with PSAC did not significantly differ between those with SRC and non-SRC over time, PSAC was increased among sports categorized as limited contact or impact. This observation suggests that certain sports activities characterized by increased velocity or risk of falls from greater heights predispose participants to a higher likelihood of persistent symptoms following SRC. This observation may also be explained by sociocultural norms in sports.^52^ The sports included in the limited-contact or limited-impact group are all individual sports where the athlete may have more autonomy. Contact or collision sports are team sports with a strong tough-it-out culture (eg, football or hockey) that encourages athletes to return to sport sooner out of fear (eg, of losing playing status). Interestingly, dodgeball, often played during school gym classes and recess, exhibited an increased percentage of PSAC over time, despite being considered a limited-contact sport. In a US cohort of individuals with dodgeball-related injuries,^53^ 25.7% of injuries involved the head and neck, and concussions accounted for 5.6% of children aged 0 to 19 years. Due to concerns over increased injury risk and bullying instances associated with dodgeball, the National Association for Sport and Physical Education issued a position statement in 2006 indicating that dodgeball was not an appropriate activity for students from kindergarten to grade 12,^54^ prompting some schools to ban dodgeball. Unlike sports that focus on scoring goals or baskets, the primary objective in dodgeball is to throw balls directly at other players, aiming to hit and eliminate them from the game. Despite these concerns, dodgeball or forms of dodgeball continue to be played in school settings during recess and physical education classes.

### Clinical Management

Data regarding the recovery trajectories of children participating in limited-contact, limited-impact, or noncontact sports, or of children who are not participating in sports and sustain a non-SRC, is limited. The present study findings suggest that clinicians can employ standardized management protocols^55^ for both SRC and non-SRC in the 5- to 12-year age group because the recovery trajectories were similar. Furthermore, the present findings underscore the possibility that managing SRC may not require distinct strategies based on sports classification. Instead, it may be more appropriate for clinicians to consider the specific dynamics of the activity, such as velocity and risk of falls from heights. This nuanced perspective can aid in assessing the likelihood of persisting symptoms.

### Strengths and Limitations

While this study represents one of the largest investigations in a pediatric population, it is constrained by several limitations. The 5P dataset, collected from 2013 to 2015, predates the current recommendation of returning to activities 48 hours postinjury.^56,57^ However, this limitation is unlikely to significantly impact recovery trajectories, except potentially resulting in faster recovery among children. Our sampling was restricted to EDs, potentially biasing our sample toward children with higher initial symptom burdens or more severe injuries. Our inclusion criteria, requiring 85% completeness of the PCSI at each time point, may have introduced bias by not distinguishing between participants who dropped out entirely from the study, those with missing data at specific time points, or those who simply did not complete enough PCSI items. The definition of recovery based on self- or parent-rated symptoms may have limited the representativeness of our findings because they do not capture neurophysiological or neuropsychological recovery. Furthermore, self-report measures are subjective and susceptible to bias. Additionally, this study did not differentiate between athletes and nonathletes regarding the level of medical care. An athlete might have experienced a non-SRC, while a nonathlete could have had an SRC. Future research with detailed data on preinjury sports characteristics and medical care access is needed to better understand how these factors may impact concussion outcomes.

## Conclusions

In this cohort study of children aged 5 to 12 years with an acute SRC or non-SRC, symptom recovery trajectories over time were similar in both age groups (5-7 years and 8-12 years). Furthermore, no differences between symptom trajectories were found between contact or collision, limited-contact, and noncontact sports. The current findings suggest that management protocols for SRC and non-SRC (excluding motor vehicle crashes and assaults) can be similar for younger populations because they exhibit comparable patterns of symptom improvement over time.

## Notes

Supplement 1. **eTable 1.** Examples of Sport and Non-Sport Activities That Can Lead to Concussions **eTable 2.** Sport Classification **eTable 3.** Missing PCSI Outcome at Each Time Point **eFigure 1.** Participant Inclusion Flowchart **eTable 4.** Personal Characteristics by Type of Contact/Collision **eTable 5.** Association Between Symptom Trajectories and Time Stratified by Sport-Related Concussion (SRC) and Non-SRC in Children Aged 5-7 (N=477) **eTable 6.** Association Between Symptom Trajectories and Time Stratified by Sport-Related Concussion (SRC) and Non-SRC in Children Aged 8-12 (N=1,157) **eTable 7.** Association Between Symptom Trajectories and Time Stratified by Sport Classification for Children Aged 5-7 **eTable 8.** Association Between Symptom Trajectories and Time Stratified by Sport Classification for Children Aged 8-12 **eTable 9**. Association between Persisting Symptoms After Concussion and Sport Classification for Children Aged 5-7 **eTable 10.** Contrasts for the Simple Effect of Sport Classification in the Association Between Persisting Symptoms After Concussion and Sport Classification for Children Aged 5-7 **eTable 11.** Association Between Persisting Symptoms After Concussion and Sport Classification for Children Aged 8-12 **eTable 12.** Contrasts for the Simple Effect of Sport Classification in the Association Between Persisting Symptoms After Concussion and Sport Classification for Children Aged 8-12 **eFigure 2.** Proportion of Persisting Symptoms After a Concussion (≥3 Symptoms) Over Time With at Least 10 Patients in Each Sport

Supplement 2.
Data Sharing Statement
