# Supplementary material for: Symptom Recovery in Children Aged 5 to 12 Years With Sport-Related and Non–Sport-Related Concussion
Source: JAMA Netw Open. 2024 Dec 4;7(12):e2448797. doi: 10.1001/jamanetworkopen.2024.48797 (PMC11618468; doi:10.1001/jamanetworkopen.2024.48797)
Supplement: Supplement 1. — eTable 1. Examples of Sport and Non-Sport Activities That Can Lead to Concussions eTable 2. Sport Classification eTable 3. Missing PCSI Outcome at Each Time Point eFigure 1. Participant Inclusion Flowchart eTable 4. Personal Characteristics by Type of Contact/Collision eTable 5. Association Between Symptom Trajectories and Time Stratified by Sport-Related Concussion (SRC) and Non-SRC in Children Aged 5-7 (N=477) eTable 6. Association Between Symptom Trajectories and Time Stratified by Sport-Related Concussion (SRC) and Non-SRC in Children Aged 8-12 (N=1,157) eTable 7. Association Between Symptom Trajectories and Time Stratified by Sport Classification for Children Aged 5-7 eTable 8. Association Between Symptom Trajectories and Time Stratified by Sport Classification for Children Aged 8-12 eTable 9. Association between Persisting Symptoms After Concussion and Sport Classification for Children Aged 5-7 eTable 10. Contrasts for the Simple Effect of Sport Classification in the Association Between Persisting Symptoms After Concussion and Sport Classification for Children Aged 5-7 eTable 11. Association Between Persisting Symptoms After Concussion and Sport Classification for Children Aged 8-12 eTable 12. Contrasts for the Simple Effect of Sport Classification in the Association Between Persisting Symptoms After Concussion and Sport Classification for Children Aged 8-12 eFigure 2. Proportion of Persisting Symptoms After a Concussion (≥3 Symptoms) Over Time With at Least 10 Patients in Each Sport [file jamanetwopen-e2448797-s001.pdf]

Supplemental Online Content

Ledoux A-A, Sicard V, Bijelic V, et al; Pediatric Emergency Research Canada (PERC) Predicting and Preventing Postconcussive Problems in Pediatrics (5P) Concussion Team. Symptom recovery in children aged 5 to 12 years with sport-related and non–sport-related concussion. *JAMA Netw. Open*. 2024;7(12):e2448797. doi:10.1001/ jamanetworkopen.2024.48797

eTable 1. Examples of Sport and Non-Sport Activities That Can Lead to Concussions
eTable 2. Sport Classification
eTable 3. Missing PCSI Outcome at Each Time Point
eFigure1. Participant Inclusion Flowchart
eTable 4. Personal Characteristics by Type of Contact/Collision
eTable 5. Association Between Symptom Trajectories and Time Stratified by Sport-Related Concussion (SRC) and Non-SRC in Children Aged 5-7 (N=477)
eTable 6. Association Between Symptom Trajectories and Time Stratified by Sport-Related Concussion (SRC) and Non-SRC in Children Aged 8-12 (N=1,157)
eTable 7. Association Between Symptom Trajectories and Time Stratified by Sport Classification for Children Aged 5-7
eTable 8. Association Between Symptom Trajectories and Time Stratified by Sport Classification for Children Aged 8-12
eTable 9. Association between Persisting Symptoms After Concussion and Sport Classification for Children Aged 5-7
eTable 10. Contrasts for the Simple Effect of Sport Classification in the Association Between Persisting Symptoms After Concussion and Sport Classification for Children Aged 5-7
eTable 11. Association Between Persisting Symptoms After Concussion and Sport Classification for Children Aged 8-12
eTable 12. Contrasts for the Simple Effect of Sport Classification in the Association Between Persisting Symptoms After Concussion and Sport Classification for Children Aged 8-12
eFigure 2. Proportion of Persisting Symptoms After a Concussion (≥3 Symptoms) Over Time With at Least 10 Patients in Each Sport

This supplemental material has been provided by the authors to give readers additional information about their work.

© 2024 Ledoux A-A et al. *JAMA Network Open*.

eTable 1. Examples of Sport and Non-Sport Activities That Can Lead to Concussions

Sport activities	Non-sport activities/falls	Excluded from this analysis
• Football<br>• Soccer<br>• Basketball<br>• Cheerleading<br>• Track and field<br>• Dodgeball<br>• Kickball<br>• Recreational play/recess<br>• Tobogganing/sledding<br>• Snowboarding/skiing<br>• Bicycling<br><br>* *See eTable2 for list of sports included in the current study*	• Slipping on ice or wet surfaces<br>• Falling from stairs or furniture<br>• Tripping and falling while walking<br>• Bumping into doors, walls or furniture<br>• Walking into low-hanging objects<br>• Colliding accidentally with a person<br>• Dropping an object on their head	• MVC: Car, motorcycle, or ATV accidents<br>• Assaults or physical altercations

Notes. ATV=All-Terrain Vehicles; MVC=Motor Vehicle Crashes. These examples represent potential causes of sports and non-sports concussions. While they illustrate possible scenarios, they may not necessarily reflect the specific causes of concussions in the current study. This list is not exhaustive.

eTable 2. Sport Classification

Contact/Collision	Limited Contact/Impact	Non-contact
• Hockey<br>• Football<br>• Soccer<br>• Rugby<br>• Lacrosse<br>• Ringette<br>• Roller derby<br>• Combat sports (Karate, Judo, TKD, MMA, Boxing)<br>• Wrestling	• Bicycling<br>• Horseback riding<br>• Skateboarding/rollerblading<br>• Scooter<br>• Basketball<br>• Volleyball<br>• Baseball/softball<br>• Dodgeball<br>• Handball<br>• Tchuk ball<br>• Floor/street hockey<br>• Field hockey<br>• Trampoline<br>• Gymnastics<br>• Cheerleading<br>• Ski/snowboarding<br>• Skating<br>• Tobogganing/sledding<br>• Luge<br>• Skeleton<br>• Synchronized swimming<br>• Waterpolo<br>• Watersport (wakeboard)<br>• Parkour<br>• Ultimate Frisbee	• Recreational play (gym, recess)<br>• Swimming<br>• Badminton<br>• Tennis<br>• Flag football/touch<br>• Dance<br>• Curling<br>• Athletics/track<br>• Earth ball<br>• Rock climbing<br>• Hiking<br>• Sailing<br>• Bowling<br>• Golf<br>• Strength training<br>• Cross country skiing

Notes. MMA=Mixed Martial Arts; TKD=Tae Kwon Do.

© 2024 Ledoux A-A et al. *JAMA Network Open.*

eTable 3. Missing PCSI Outcome at Each Time Point							
Age group 5-7					Age group 8-12		
**Week**	**Total**	**Missing PCSI (N)**	**Missing PCSI (%)**		**Total**	**Missing PCSI (N)**	**Missing PCSI (%)**
0	513	19	3.70		1234	30	2.43
1	513	72	14.04		1234	174	14.10
2	513	72	14.04		1234	175	14.18
4	513	85	16.57		1234	183	14.83
8	513	104	20.27		1234	248	20.10
12	513	128	24.95		1234	308	24.96

Notes. PCSI=Post-Concussion Symptom Inventory

© 2024 Ledoux A-A et al. *JAMA Network Open*.

eFigure1. Participant Inclusion Flowchart

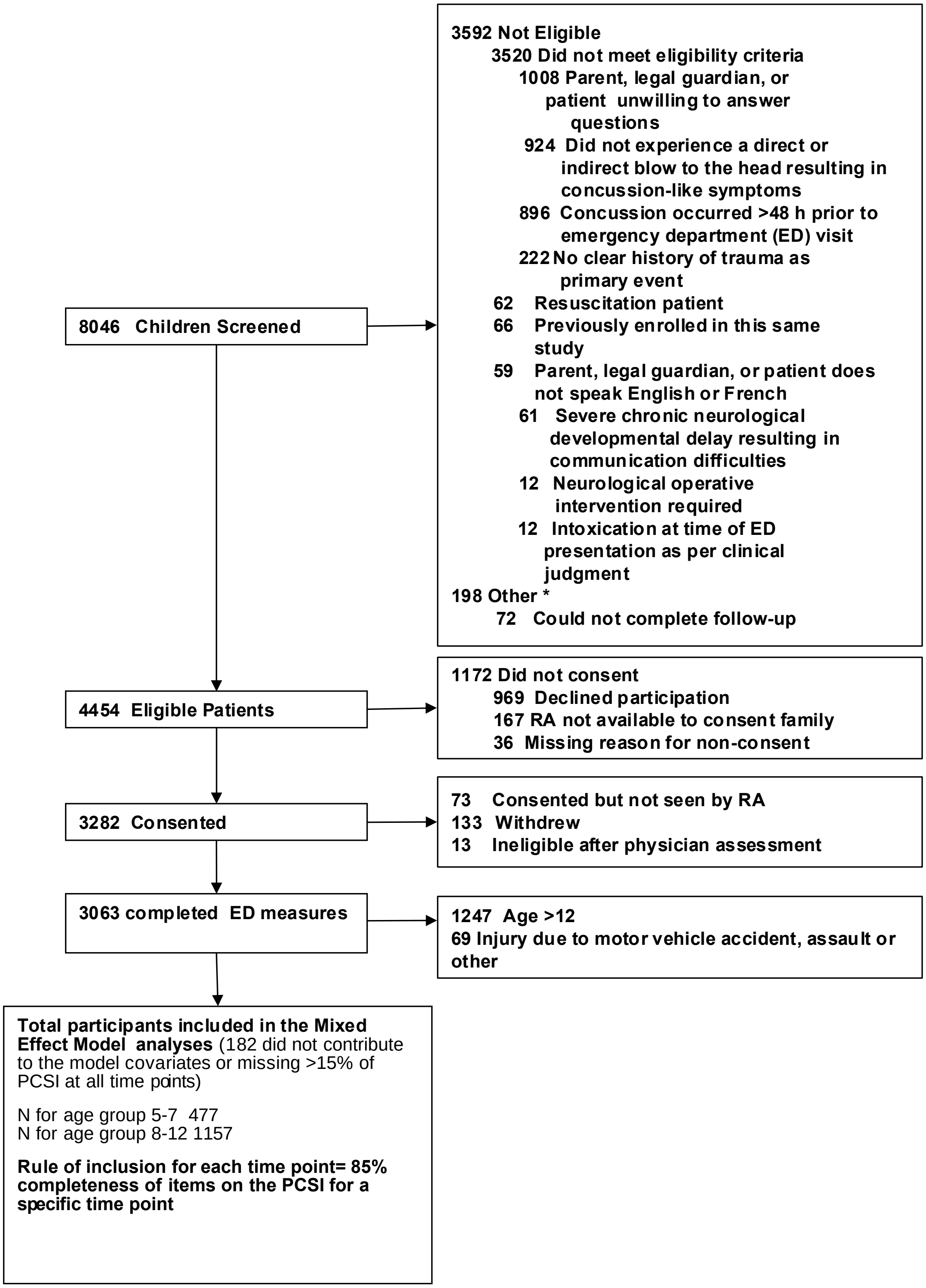

ED=Emergency Department; RA=Research Assistant; PCSI=Post-Concussion Symptom Inventory.

© 2024 Ledoux A-A et al. *JAMA Network Open*.

eTable 4. Personal Characteristics by Type of Contact/Collision

Variable	Age 5-7 years			Age 8-12 years		
	**Non-Contact** N = 111	**Limited Contact** N = 44	**Collision** N = 52	**Non-Contact** N = 176[1]	**Limited Contact** N = 229	**Collision** N = 385
**Age**	6.67 (0.82)	6.48 (0.88)	6.86 (0.83)	10.32 (1.37)	10.94 (1.48)	10.88 (1.33)
**Sex**						
Male	71 (64%)	27 (61%)	44 (85%)	105 (60%)	123 (54%)	319 (83%)
Female	40 (36%)	17 (39%)	8 (15%)	71 (40%)	106 (46%)	66 (17%)
**Prior concussion and symptom duration**						
No prior concussion; symptom duration <1 wk	107 (96%)	43 (98%)	51 (98%)	162 (92%)	216 (94%)	358 (93%)
Prior concussion; symptom duration ≥1 wk	4 (3.6%)	1 (2.3%)	1 (1.9%)	14 (8.0%)	13 (5.7%)	27 (7.0%)
**Personal history of migraine**	7 (6.3%)	2 (4.5%)	1 (1.9%)	12 (6.8%)	25 (11%)	39 (10%)
**History of learning disability**	4 (3.6%)	3 (6.8%)	2 (3.8%)	15 (8.5%)	20 (8.7%)	22 (5.7%)
**History of attention deficit disorder**	6 (5.4%)	6 (14%)	2 (3.8%)	19 (11%)	21 (9.2%)	31 (8.1%)
**History of anxiety**	1 (0.9%)	4 (9.1%)	2 (3.8%)	16 (9.1%)	16 (7.0%)	17 (4.4%)
**History of depression**	0 (0%)	0 (0%)	0 (0%)	3 (1.7%)	2 (0.9%)	4 (1.0%)
**Loss consciousness**	0.18 (0.84)	0.11 (0.43)	0.16 (0.80)	0.08 (0.43)	0.14 (0.77)	0.04 (0.29)
**Seizure following injury**	2 (1.8%)	1 (2.3%)	2 (3.8%)	2 (1.1%)	4 (1.7%)	8 (2.1%)
**ACE - Appears dazed and confused**	37 (33%)	24 (55%)	20 (38%)	85 (48%)	112 (49%)	193 (50%)
**ACE - Answers questions slowly**	37 (33%)	14 (32%)	20 (38%)	74 (42%)	95 (41%)	159 (41%)
**ACE - Repeats questions**	15 (14%)	2 (4.5%)	7 (13%)	19 (11%)	39 (17%)	34 (8.8%)
**ACE - Forgetful of recent information**	20 (18%)	5 (11%)	5 (9.6%)	32 (18%)	49 (21%)	59 (15%)
**Wore helmet**	0 (0%)	12 (27%)	14 (27%)	2 (1.1%)	81 (35%)	237 (62%)
**Wore mouthguard**	2 (1.8%)	0 (0%)	8 (15%)	2 (1.1%)	3 (1.3%)	140 (36%)
**School days missed over past 6 months for any reason**	1 (1)	1 (1)	1 (1)	1 (1)	2 (1)	1 (1)
**Cognitive score from the Child-SCAT-3**	19.4 (5.5)	17.4 (6.6)	19.8 (4.9)	24.8 (3.5)	24.8 (3.4)	25.2 (3.0)
**BESS tandem stance # errors**	4.1 (3.6)	5.5 (3.6)	5.0 (3.9)	3.5 (3.6)	3.8 (3.7)	3.9 (3.5)

© 2024 Ledoux A-A et al. *JAMA Network Open*.

eTable 4. Personal Characteristics by Type of Contact/Collision

Variable	Age 5-7 years			Age 8-12 years		
	**Non-Contact** N = 111	**Limited Contact** N = 44	**Collision** N = 52	**Non-Contact** N = 176[1]	**Limited Contact** N = 229	**Collision** N = 385
**Normal neck range**	89 (95%)	33 (94%)	41 (93%)	153 (97%)	193 (97%)	330 (97%)
NA	17	9	8	18	31	45
**Tenderness of neck**	12 (13%)	4 (11%)	4 (9.1%)	27 (17%)	42 (21%)	78 (23%)
NA	17	9	8	18	31	47

Notes. ACE=Acute Concussion Evaluation; BESS= Balance Error Scoring System; Child-SCAT-3= Child-Sport Concussion Assessment Tool-3.

© 2024 Ledoux A-A et al. *JAMA Network Open*.

eTable 5. Association Between Symptom Trajectories and Time Stratified by Sport-Related Concussion (SRC) and Non-SRC in Children Aged 5-7 (N=477)

Characteristic	Beta	95% CI[1]	p-value
Type of injury			0.4
Non-sport/Fall	—	—	
Sports/Recreation	1.40	-1.90, 4.70	0.4
Age	0.15	-0.21, 0.51	0.4
**Time**			**<0.001**
rcs(time, parms = my.knots)time	-2.10	-2.30, -1.90	<0.001
rcs(time, parms = my.knots)time'	6.20	5.60, 6.90	<0.001
Type of collision			0.6
Non-Contact	—	—	
Limited Contact	-0.05	-0.90, 0.81	>0.9
Collision	-0.39	-1.20, 0.40	0.3
Prior concussion and symptom duration			0.2
No prior concussion; symptom duration <1 week	—	—	
Prior concussion; symptom duration ≥1 week	0.75	-0.44, 1.90	0.2
Personal history of migraine	0.25	-0.71, 1.20	0.6
History of learning disability	-1.00	-2.20, 0.15	0.09
History of attention deficit disorder	0.79	-0.28, 1.80	0.15
History of anxiety	-0.43	-1.80, 0.91	0.5
Loss of consciousness duration	0.03	-0.36, 0.42	0.9
Seizure following injury	0.25	-1.30, 1.80	0.8
**ACE - Appears dazed and confused**	0.68	0.20, 1.20	**0.01**
ACE - Answers questions slowly	0.41	-0.08, 0.91	0.10
ACE - Repeats questions	0.62	-0.10, 1.30	0.09
ACE - Forgetful of recent information	-0.06	-0.66, 0.53	0.8
Wore helmet	-0.39	-1.50, 0.75	0.5
Wore mouthguard	-0.08	-1.70, 1.60	>0.9
**School days missed over past 6 months for any reason**	0.24	0.03, 0.45	**0.03**
Child-SCAT3	-0.01	-0.05, 0.03	0.7
**BESS tandem stance # errors**	0.06	0.00, 0.11	**0.04**
Type of injury * Age			0.5
Sports/Recreation * Age	-0.16	-0.65, 0.33	0.5
Type of injury * Time			0.8
Sports/Recreation * rcs(time, parms = my.knots)time	0.01	-0.29, 0.30	>0.9
Sports/Recreation * rcs(time, parms = my.knots)time'	-0.09	-1.10, 0.92	0.9

Notes. ACE=Acute Concussion Evaluation; BESS=Balance Error Scoring System; CI=Confidence Interval; SCAT3= Sport Concussion Assessment Tool version 3. Bold denotes significance.

© 2024 Ledoux A-A et al. *JAMA Network Open*.

eTable 6. Association Between Symptom Trajectories and Time Stratified by Sport-Related Concussion (SRC) and Non-SRC in Children Aged 8-12 (N=1,157)

**Characteristic**	**Beta**	**95% CI[1]**	**p-value**
Type of injury			0.3
Non-sport/Fall	—	—	
Sports/Recreation	-1.60	-4.80, 1.70	0.3
Age	0.23	-0.03, 0.49	0.087
**Time**			**<0.001**
rcs(time, parms = my.knots)time	-2.6	-2.8, -2.4	<0.001
rcs(time, parms = my.knots)time'	15	14, 16	<0.001
Type of collision			0.5
Non-Contact	—	—	
Limited Contact	0.17	-0.51, 0.86	0.6
Collision	-0.20	-0.88, 0.49	0.6
Prior concussion and symptom duration			0.06
No prior concussion; symptom duration <1 wk	—	—	
Prior concussion; symptom duration ≥1 wk	0.73	-0.03, 1.50	0.06
Personal history of migraine	0.36	-0.27, 1.00	0.3
History of learning disability	-0.50	-1.30, 0.28	0.2
History of attention deficit disorder	-0.17	-0.90, 0.57	0.7
**History of anxiety**	1.10	0.26, 1.90	**0.01**
History of depression	-1.50	-3.5, 0.54	0.2
**Loss of consciousness duration**	0.41	0.09, 0.73	**0.01**
Seizure following injury	1.40	-0.14, 2.90	0.08
**ACE - Appears dazed and confused**	0.68	0.24, 1.10	**0.002**
**ACE - Answers questions slowly**	0.78	0.33, 1.20	**<0.001**
**ACE - Repeats questions**	0.79	0.14, 1.40	**0.02**
**ACE - Forgetful of recent information**	0.76	0.21, 1.30	**0.007**
Wore helmet	0.06	-0.54, 0.67	0.8
Wore mouthguard	-0.30	-1.0, 0.44	0.4
**School days missed over past 6 months for any reason**	**0.29**	**0.09, 0.48**	**0.004**
**Child-SCAT3**	**-0.08**	**-0.14, -0.02**	**0.008**
**BESS tandem stance # errors**	**0.16**	**0.10, 0.21**	**<0.001**
Type of injury * Age			0.4
Sports/Recreation * Age	0.14	-0.17, 0.44	0.4
Type of injury * Time			>0.9
Sports/Recreation * rcs(time, parms = my.knots)time	-0.01	-0.23, 0.21	>0.9
Sports/Recreation * rcs(time, parms = my.knots)time'	0.11	-1.50, 1.70	0.9

Notes. ACE=Acute Concussion Evaluation; BESS=Balance Error Scoring System; CI=Confidence Interval; SCAT3= Sport Concussion Assessment Tool version 3. Bold denotes significance.

© 2024 Ledoux A-A et al. *JAMA Network Open.*

eTable 7. Association Between Symptom Trajectories and Time Stratified by Sport Classification for Children Aged 5-7

Characteristic	$N_{obs.}$	Beta	95% CI[1]	p-value
Age	1,090	-0.06	-0.52, 0.39	0.8
Type of collision	1,090			0.6
Non-Contact		—	—	
Limited Contact		-0.50	-1.70, 0.68	0.4
Collision		-0.36	-1.40, 0.73	0.5
**Time**	1,090			**<0.001**
rcs(time, parms = my.knots)time		-2.0	-2.30, -1.70	<0.001
rcs(time, parms = my.knots)time'		6.50	5.40, 7.50	<0.001
Prior concussion and symptom duration	1,090			0.3
No prior concussion; symptom duration <1 wk		—	—	
Prior concussion; symptom duration ≥1 wk		0.95	-0.99, 2.90	0.3
Personal history of migraine	1,090	0.25	-1.40, 1.90	0.8
**History of learning disability**	1,090	-1.90	-3.70, -0.09	**0.04**
**History of attention deficit disorder**	1,090	1.50	0.01, 3.00	**0.05**
History of anxiety	1,090	-0.43	-2.30, 1.50	0.7
Loss of consciousness duration	1,090	0.07	-0.37, 0.51	0.8
Seizure following injury	1,090	0.75	-1.40, 2.90	0.5
ACE - Appears dazed and confused	1,090	0.49	-0.33, 1.30	0.2
**ACE - Answers questions slowly**	1,090	1.10	0.20, 1.90	**0.02**
ACE - Repeats questions	1,090	1.00	-0.09, 2.20	0.07
ACE - Forgetful of recent information	1,090	-0.63	-1.70, 0.39	0.2
Wore helmet	1,090	-0.58	-1.90, 0.68	0.4
Wore mouthguard	1,090	-0.08	-1.90, 1.80	>0.9
School days missed over past 6 months for any reason	1,090	0.24	-0.12, 0.60	0.2
Child-SCAT3	1,090	0.01	-0.06, 0.08	0.7
BESS tandem stance # errors	1,090	0.04	-0.05, 0.14	0.4
Type of collision * Time	1,090			0.2
Limited Contact * rcs(time, parms = my.knots)time		0.36	-0.17, 0.90	0.2
Collision * rcs(time, parms = my.knots)time		-0.21	-0.71, 0.29	0.4
Limited Contact * rcs(time, parms = my.knots)time'		-1.20	-3.20, 0.87	0.3
Collision * rcs(time, parms = my.knots)time'		1.10	-0.85, 3.00	0.3

Notes. ACE=Acute Concussion Evaluation; BESS=Balance Error Scoring System; CI=Confidence Interval; SCAT3= Sport Concussion Assessment Tool version 3. Bold denotes significance.

© 2024 Ledoux A-A et al. *JAMA Network Open*.

eTable 8. Association Between Symptom Trajectories and Time Stratified by Sport Classification for Children Aged 8-12

Characteristic	$N_{obs.}$	Beta	95% CI[1]	p-value
**Age**	4,083	0.32	0.15, 0.50	**<0.001**
Type of collision	4,083			0.5
Non-Contact		—	—	
Limited Contact		0.45	-0.41, 1.30	0.3
Collision		0.16	-0.68, 0.99	0.7
**Time**	4,083			**<0.001**
rcs(time, parms = my.knots)time		-2.60	-2.90, -2.40	<0.001
rcs(time, parms = my.knots)time'		16	14, 19	<0.001
**Prior concussion and symptom duration**	4,083			**0.02**
No prior concussion; symptom duration <1 wk		—	—	
Prior concussion; symptom duration ≥1 wk		1.10	0.15, 2.00	0.02
Personal history of migraine	4,083	-0.08	-0.85, 0.70	0.8
History of learning disability	4,083	0.08	-0.88, 1.00	0.9
History of attention deficit disorder	4,083	-0.49	-1.40, 0.38	0.3
**History of anxiety**	4,083	1.60	0.59, 2.6	**0.002**
History of depression	4,083	-0.19	-2.50, 2.20	0.9
Loss of consciousness duration	4,083	0.41	-0.13, 0.95	0.14
Seizure following injury	4,083	1.70	-0.07, 3.40	0.06
**ACE - Appears dazed and confused**	4,083	0.74	0.22, 1.30	**0.006**
**ACE - Answers questions slowly**	4,083	0.68	0.14, 1.20	0.01
ACE - Repeats questions	4,083	0.58	-0.17, 1.30	0.13
**ACE - Forgetful of recent information**	4,083	0.74	0.07, 1.40	**0.03**
Wore helmet	4,083	0.00	-0.60, 0.59	>0.9
Wore mouthguard	4,083	-0.24	-0.98, 0.50	0.5
**School days missed over past 6 months for any reason**	4,083	0.27	0.03, 0.51	**0.03**
Child-SCAT3	4,083	-0.04	-0.12, 0.04	0.3
**BESS tandem stance # errors**	4,083	0.14	0.07, 0.21	**<0.001**
Type of collision * Time	4,083			0.4
Limited Contact * rcs(time, parms = my.knots)time		0.03	-0.33, 0.38	0.9
Collision * rcs(time, parms = my.knots)time		-0.06	-0.39, 0.26	0.7
Limited Contact * rcs(time, parms = my.knots)time'		-0.78	-3.50, 2.00	0.6
Collision * rcs(time, parms = my.knots)time'		-0.02	-2.50, 2.50	>0.9

Notes. ACE=Acute Concussion Evaluation; BESS=Balance Error Scoring System; CI=Confidence Interval; SCAT3= Sport Concussion Assessment Tool version 3. Bold denotes significance.

© 2024 Ledoux A-A et al. *JAMA Network Open.*

eTable 9. Association between Persisting Symptoms After Concussion and Sport Classification for Children Aged 5-7

Characteristic	$N_{obs.}$	log(OR)	95% CI	p-value
**Type of collision**				**0.04**
Non-Contact	1,687	—	—	
Limited Contact	194	0.66	-0.15, 1.5	
Collision	222	-0.74	-1.6, 0.08	
**Time**	2,103	-0.17	-0.21, -0.13	**<0.001**

Notes. OR=Odds Ratio. CI=Confidence Interval. Bold denotes significance.

eTable 10. Contrasts for the Simple Effect of Sport Classification in the Association Between Persisting Symptoms After Concussion and Sport Classification for Children Aged 5-7

Contrast	Estimate	SE	p-value
(Non-Contact) – Limited Contact	-0.66	0.41	0.25
(Non-Contact) – Contact/Collision	0.74	0.42	0.18
**Limited Contact – Contact/Collision**	1.40	0.56	**0.03**

Notes. SE=Standard Error. Bold denotes significance after adjusting for multiple comparisons using the Tukey method.

eTable 11. Association Between Persisting Symptoms After Concussion and Sport Classification for Children Aged 8-12

Characteristic	$N_{obs.}$	log(OR)	95% CI	p-value
**Type of collision**				**0.05**
Non-Contact	2,379	—	—	
Limited Contact	1,013	0.43	-0.01, 0.86	
Collision	1,670	-0.15	-0.52, 0.22	
**Time**	5,062	-0.27	-0.30, -0.25	**<0.001**

Notes. OR=Odds Ratio. CI=Confidence Interval. Bold denotes significance.

eTable 12. Contrasts for the Simple Effect of Sport Classification in the Association Between Persisting Symptoms After Concussion and Sport Classification for Children Aged 8-12

Contrast	Estimate	SE	p-value
(Non-Contact) – Limited Contact	-0.43	0.22	0.13
(Non-Contact) – Contact/Collision	0.15	0.19	0.71
**Limited Contact – Contact/Collision**	0.57	0.24	**0.04**

Notes. SE=Standard Error. Bold denotes significance after adjusting for multiple comparisons using the Tukey method.

© 2024 Ledoux A-A et al. *JAMA Network Open.*

eFigure 2. Proportion of Persisting Symptoms After a Concussion (≥3 Symptoms) Over Time With at Least 10 Patients in Each Sport

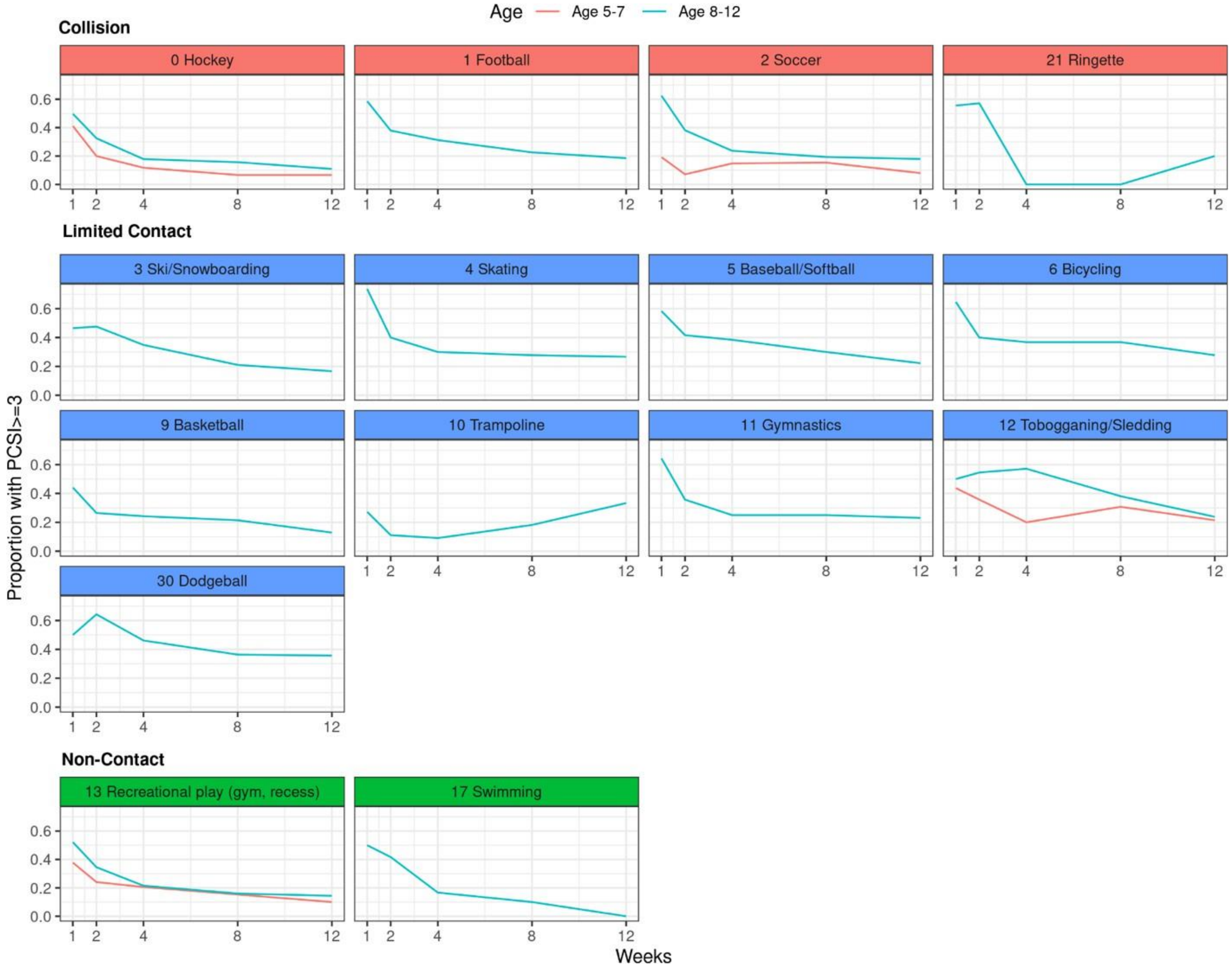

PCSI=Post-Concussion Symptom Inventory. Red indicates the 5-7 age group. Blue indicates the 8-12 age group.

© 2024 Ledoux A-A et al. *JAMA Network Open*.
