# Supplementary material for: Symptom Recovery in Children Aged 5 to 12 Years With Sport-Related and Non–Sport-Related Concussion
Source: JAMA Netw Open. 2024 Dec 4;7(12):e2448797. doi: 10.1001/jamanetworkopen.2024.48797 (PMC11618468; doi:10.1001/jamanetworkopen.2024.48797)
Supplement: Supplement 2. — Data Sharing Statement [file jamanetwopen-e2448797-s002.pdf]

Data Sharing Statement

Ledoux. Symptom Recovery in Children Aged 5 to 12 Years With Sport-Related and Non–Sport-Related Concussion. *JAMA Netw Open*. Published December 04, 2024. doi:10.1001/jamanetworkopen.2024.48797

Data
Data available: Yes
Data types: Deidentified participant data
How to access data: To obtain de-identified data please access the 5P: Predicting Persistent Postconcussive Problems in Pediatrics database via Brain-Code: https://www.braincode.ca/
When available: With publication

Supporting Documents
Document types: None

Additional Information
Who can access the data: To obtain de-identified data please access the 5P: Predicting Persistent Postconcussive Problems in Pediatrics database via Brain-Code: https://www.braincode.ca/
Types of analyses: To obtain de-identified data please access the 5P: Predicting Persistent Postconcussive Problems in Pediatrics database via Brain-Code: https://www.braincode.ca/
Mechanisms of data availability: To obtain de-identified data please access the 5P: Predicting Persistent Postconcussive Problems in Pediatrics database via Brain-Code: https://www.braincode.ca/
